# Does unemployment in family affect pregnancy outcome in conditions of high quality maternity care?

**DOI:** 10.1186/1471-2458-6-46

**Published:** 2006-02-24

**Authors:** Kaisa Raatikainen, Nonna Heiskanen, Seppo Heinonen

**Affiliations:** 1 Department of Obstetrics and Gynaecology, Kuopio University Hospital, P.O.Box 1777, 70211 Kuopio, Finland

## Abstract

**Background:**

The influence of unemployment in the family on pregnancy outcome is controversial. Only a few studies have involved investigation of the effect of unemployment of the father on pregnancy. The objective of this study was to assess the effects of unemployment of one or both parents on obstetric outcome in conditions of free antenatal care attended by the entire pregnant population.

**Methods:**

The data of 24 939 pregnancies included maternal risk factors, pregnancy characteristics and outcome, and was based on a self administered questionnaire at 20 weeks of pregnancy and on clinical records.

**Results:**

Unemployment was associated with adolescent maternal age, unmarried status and overweight, anemia, smoking, alcohol consumption and prior pregnancy terminations. Multivariate logistic regression analysis indicated that after controlling for these maternal risk factors small differences only were found in pregnancy outcomes between unemployed and employed families. Unemployed women had significantly more often small-for-gestational-age (SGA) infants, at an OR of 1.26 (95% CI: 1.12 – 1.42) whereas, in families where both parents were unemployed, the risk of SGA was even higher at an OR of 1.43 (95% CI: 1.18 – 1.73). Otherwise, pregnancy outcome was comparable in the groups studied.

**Conclusion:**

Free antenatal care was unable to fully overcome the adverse pregnancy outcomes associated with unemployment, SGA risk being highest when both parents are unemployed.

## Background

Unemployment is strongly associated with an increased risk of morbidity and mortality. Unemployed persons use more general health services, have more physical and mental health problems and even have a higher suicide rate than their employed counterparts. Lower levels of psychological well-being have been systematically found in all studies – at all ages and in both sexes [1,2].

The topic of unemployment and pregnancy outcome is of interest for several reasons, since it is a marker of socioeconomic status, a potential marker of stress, an indicator of poor physical or mental health, a proxy for chemical exposures like alcohol or cigarette smoke etc.

Much controversy exists in the literature with regard to the influence of unemployment in the family on pregnancy outcome. Some investigators have shown associations with preterm delivery [3-5], low birth weight [5] and a higher perinatal mortality rate [5], whereas others have shown opposite results [6-8]. However, there appears to be consensus that unemployment in pregnancy shows a strong association with social disadvantage, low income, being unmarried and having unfavorable health behaviors. The correlation between unemployment and ill health has been explained as a result of both exposure to these factors and selection of unhealthy persons to be unemployed. The relationship is complex and causation cannot easily be proved [9].

Only a few studies have involved investigation of the effect of unemployment of the father on pregnancy. These studies have shown a change in maternal health behavior, but interestingly no association with low birth weight or preterm delivery [10]. In Finland maternity care is provided free of charge and is used by virtually the entire (99.7%) pregnant population, the first visit at maternity care takes places at average of 9.7 weeks of pregnancy and the average number of visits to maternity care during pregnancy is 17.3 [11]. The opportunity to receive maternity care during pregnancy is not affected by the economic situation of a family and this kind of antenatal care is rare even in European countries, in other Scandinavian countries maternity care is comparable to Finland.

The aim of this study was to assess the effects of unemployment of one or both parents on obstetric risk factors and pregnancy outcome in conditions of free, high standard maternity care, used by almost the entire pregnant population, to gain more understanding of whether the poor pregnancy outcomes associated with unemployment in family are avoidable in these conditions.

## Methods

We investigated the total population at Kuopio University Hospital who gave birth between January 1989 and December 1999, a total of 25 679 pregnancies. Kuopio University Hospital is a university teaching hospital and the only hospital in Kuopio District offering obstetric care. Of the study population, 0.76% did not attend antenatal care of any kind before they were in birth. Unemployment rate of women in childbearing age in Kuopio district varied during the period of time concerned between 3.0% and 15.1%, whereas the equivalent figures for Finland varied between 3.2% and 16.6%. Economical depression that was experienced in Finland in early 1990's can readily be seen in the actual numbers of women unemployed [12]. Data from 3388 women unemployed during pregnancy, (study group I), 1551 women whose partner was unemployed during pregnancy (study group II) and 1037 women who were unemployed and whose partner was also unemployed during pregnancy (study group III). The reference population (no parental unemployment) consisted of 18 963 women. Multiple pregnancies (n = 484, 1.88% of all pregnancies) and major fetal structural anomalies (31 in study group I, 16 in study group II, 13 in study group III and 196 in the reference group, totaling 1.0% of all viable pregnancies) were excluded since these pregnancies carry an unusually high risk of adverse outcome, and the effect of unemployment on these pregnancies would be difficult to distinguish. After exclusions, a total of 24 939 pregnancies were analyzed. 

Our database included information on maternal characteristics, based on information from a self-administered questionnaire at 20 weeks of pregnancy and completed by nurse interviews at visits to Kuopio University Hospital. The Institutional Review Board has accepted the study and childbearing women have given informed consent at the time of data collection and patient data has been processed anonymously. The questionnaire consisted of over 50 questions concerning marital status, employment data, paternal characteristics, previous operations, illnesses and obstetric history, contraceptive use and smoking and alcohol consumption. The information on pregnancy complications, pregnancy outcome and neonatal period was based on clinical records, collected to the database by the team who took care of the delivery and neonatal care. Unemployment status is clearly distinguishable from that of housewives, who are not entitled to unemployment benefits when they are not actively seeking a job: a multiple choice question concerning profession included separate options for both housewives and unemployed women. Unmarried women were classified according their unemployment status. The estimation of gestational age was based on menstrual history and ascertained by measuring fetal crowd rump length by ultrasound at 10 to 12 weeks of pregnancy.

The following definitions were used: preterm birth, delivery before 37 weeks of gestation; prolonged gravidity, delivery after 42 weeks of gestation; pre-eclampsia, repeated blood pressure measurement exceeding 149/90 mmHg with proteinuria exceeding 0.5 g/ day. Infants were considered small for gestational age (SGA) when the age- and sex-specific birth weight was below the tenth percentile according to the normal tables for our population [13]. Grand multiparity was defined having over 7 previous deliveries. Mother was considered a smoker when she smoked more than 5 cigarettes per day during pregnancy. Low hemoglobin was defined as hemoglobin under 100 g/l. The pH limit used for fetal acidosis was 7.15 at birth. Overweight was defined as pre-gravid BMI > 25 (weight in kg divided by the square of the height in m). If a subject had two abnormalities, such as SGA and preterm delivery, each was considered an independent outcome and the subject was included in both categories.

Statistical differences between subjects and controls were evaluated by using Chi-square tests (dichotomous variables), and Fisher's exact test was applied when the minimal estimated expected value was less than five. P < 0.05 was considered statistically significant. Continuous variables were analyzed by using two-tailed, pooled *t *tests. Possible confounding variables were identified from background data, obstetric risk factors, and health behaviors. Multivariate analysis of significant or nearly significant effects (p < 0.1) of lifestyle variables concerned in this study (maternal age over 35 or under 17, being single mother, primiparity, smoking during pregnancy, history of infertility, previous miscarriages, previous induced abortions, short time or long time since previous pregnancy or using IUD before this pregnancy) was based on multiple logistic regression analysis (BMDP Statistical Software Inc., Los Angeles, CA). Confidence intervals were evaluated at 95%.

## Results

In 13.6 % of single pregnancies without major structural anomalies the mother was unemployed, in 6.2% the father and in 4.2% of pregnancies studied both parents were unemployed. Compared with the reference group, the women in groups I-III were younger: the mean maternal age (± standard deviation) in the reference group was 29.4 ± 5.2 y, vs. 27.5 ± 5.3 y in study group I, mother unemployed, (p < 0.05), 27.6 ± 5.5 years in study group II, father unemployed, (p < 0.05) and 25.3 ± 5.5 years in study group III, both parents unemployed, (p < 0.05). Pregnancies with major anomalies were excluded before statistical analyzes. The percentage of anomalies did not vary statistically significantly between study groups.

Table 1 shows the distribution of maternal risk factors. Adolescent age was more common and age over 35 years less common in unemployed women than in the reference group. Pregnancy outside marriage was also highly prevalent among unemployed families: 27% in the reference group, 43% when mother was unemployed and 58% when both parents were unemployed. Unemployed women were more likely to have had prior pregnancy terminations, to smoke and to use alcohol during pregnancy. Pregravid overweight (BMI > 25) and maternal diabetes were also more common (vs. the reference) in study groups I (mother unemployed) and III (both parents unemployed).

Table 2 summarizes the frequencies of various pregnancy and delivery complications. Only a few differences were recorded between the groups. Low hemoglobin during pregnancy was statistically more common in study groups I (mother unemployed) and III (both parents unemployed). No difference was found in the incidence of chorio amnionitis.

Table 3 shows pregnancy outcomes in the study groups I-III, before (Unadjusted OR) and after (Adjusted OR) multivariable analyses controlling for pregnancy risk factors found significant in this study (p < 0.1). SGA rate was found to be 22.7% higher in study group I (mother unemployed) than in the reference group, and 59.1% higher in study group III (both parents unemployed), respectively. The incidence of SGA was not increased in the study group II (father unemployed). The odds ratios changed only little in the multivariable analyses. On the other hand, the incidences of low Apgar scores, fetal acidosis at delivery, preterm delivery, admission rates to a neonatal unit, or fetal or neonatal death did not vary between the groups.

The mean birth weight (± SD) among newborns who were delivered at term (after 37 gestational weeks) was significantly lower (p < 0.05) in study groups I (mother unemployed) and III (both parents unemployed) (3612 g ± 490 g [reference] vs. 3580 ± 502 g and 3497 ± 506 g, respectively). In study group II (father unemployed) there was no difference in birth weight (3590 g ± 493 g) compared with the reference group. After adjusting for smoking the birth weights remained significantly lower (p < 0.02 and p < 0.0001) in study groups I and III (3622 g ± 485 g [reference] vs. 3601 g ± 495 g and 3605 g ± 496 g, respectively). In study group II there was no difference in birth weight (3525 g ± 508 g) compared with the reference group.

Table 4 provides direct comparison between families where the mother is unemployed and families, where both parents are unemployed. The main finding is that the risk of SGA is statistically significantly higher, OR 1.35 (1.10–1.65) in families where also father is unemployed.

## Discussion

We studied the impact of unemployment of one or both parents on the risk factors and outcome of pregnancy in conditions of free maternity care used by the entire pregnant population and found that there were marked differences between families with different employment status. The incidence of fetal growth restriction (SGA) was found to be higher in unemployed women and in families where both parents were unemployed, but not when only the father was unemployed.

We found marked differences in the pregnancy risk factors, unemployment showing a strong association with adolescent age during pregnancy, unmarried status during pregnancy and unfavorable health behaviors, specifically overweight, anemia, smoking, alcohol consumption and prior pregnancy terminations. All these are known risk factors of adverse obstetric outcome: smoking is the most important cause of fetal growth restriction [14] and alcohol consumption [15] during pregnancy is known to be associated with fetal growth restriction and anomalies. Anemia in the third trimester does not effect the pregnancy outcome but reflects nutritional status of the pregnant women and may impair the mothers ability to take care of the newborn [16,17]. Maternal adolescent age has been found to be associated with preterm births [18] and unmarried status [19] and prior pregnancy terminations [20] have also been reported to be associated with adverse pregnancy outcomes. By definition, distinguishing confounding factors from mediating factors between unemployment and ill health is difficult, if not impossible[21], and therefore, a pure statistical viewpoint was applied in the present study. However, pregnancy outcome measures in the groups studied were compared both before and after adjusting for these factors, to overcome the difficulty brought about the either confounding or mediating role of known obstetric risks being significantly associated with unemployment. Interestingly, adjusted and unadjusted ORs differed only little from each other in the present study.

Birth weight is the most important determinant of perinatal outcome, and fetal growth restriction remains a high risk factor of morbidity and mortality [22] Overall, the results of the present study revealed a reduction in mean birth weight of 32 g (study group I) to 115 g (study group III) and an increase in the rate of SGA infants among unemployed women, an OR 1.26 and among families with both parents unemployed at an OR of 1.43. Interestingly, the effect of the partner's unemployment on the socioeconomic circumstances in family was seen in obstetric outcome in the number of SGA infants only, although social disadvantage during pregnancy was clearly observed in their health behavior. The high prevalence of pregnancy risk factors found in this study in the unemployed families is in accordance with social and material deprivation. In addition to the social factors associated with unemployment there are common psychosocial associations, especially psychological stress, depression and low levels of practical support, resulting in adverse obstetric outcome [23] which persisted after adjustment for social and reproductive risk factors. The new and main finding of this study was that the social disadvantage brought about by unemployment was not overweighed by means of free antenatal care provided by the state.

So far, only a few studies on the influence of unemployment on pregnancy outcome have been reported and the results of these studies are controversial. The changes in pregnancy risk factors are consistent with previous observations. Unemployment has been associated with preterm delivery [4] (OR 1.92) and a weakly elevated (not statistically significant) risk of SGA. Unemployment of both parents has been reported to be associated with a double risk of a very preterm birth [3]. A higher proportion of low birth weight and pre-term infants and even a high perinatal mortality rate in unemployed women have been reported [5]. Psychological distress during pregnancy has been found to be associated with preterm delivery [23]. Peacock *et al. *reported that adverse social circumstances were associated with preterm birth, but they found no association between fetal growth retardation and psychosocial factors [24].

A number of investigators have reported conflicting results, with no statistically significant association between unemployment and adverse pregnancy outcome after adjustment for lifestyle variables [6,7,25,26]. Studies concerning the influence of the father being unemployed have revealed no significant excess of low birth weight or preterm delivery. However, major differences in maternal health behavior were found when the father was unemployed, specifically, delayed attendance at antenatal care, not attending classes for preparation for labor, not knowing the date of the last menstrual period, and smoking throughout pregnancy [10]. Stein *et al*. found an association between paternal unemployment and low birth weight, but this effect was statistically accounted for by low income[27]. In an identical manner, Morrison *et al. *investigated the impact of paternal socioeconomic status on pregnancy outcome. Before adjustment for lifestyle variables there seemed to be a connection between very low occupational status of the father and perinatal morbidity, but this diminished after further analysis [7].

On the other hand, in studies on SGA infants, lifestyle and psychosocial differences between families have remained important etiological factors of intrauterine growth retardation. SGA infants have been found to be more likely to have an unemployed father, to be of lower socioeconomic status and their mothers to have a lower level of education [28]. Mediating factors of unemployment's health consequences are postulated to be psychosocial. Explanations can be divided into four types: (1) poverty, (2) stress, lack of social support at work and lowered self-esteem, (3) health-related behavior and health attitudes, and (4) the effect of unemployment on the rest of work career and future socio-economic status. Furthermore, selection cannot be ruled out as a partial explanation: people who are unhealthy may be selected for low status occupations and thus be prone to become unemployed, so called healthy worker effect. [1,29-31].

Classification bias may be an issue in the current study. Data on employment status was obtained at 20 weeks of pregnancy and some women or their partners initially categorized as unemployed subsequently changed their status. Another bias may arise from the fact that in 1990's during economical depression pregnant women may have been more prone to become unemployed than women not planning to reproduce, but this would cause rather underestimation than overestimation of the effect of unemployment on reproductive health. During high unemployment, adverse pregnancy outcomes may also be seen as societal level effects, in addition to the individual effects in unemployed families.[32] The application of our findings may be limited because of differences in maternity care between countries.

## Conclusion

These results confirm those of a number of previous studies and suggest that although free-of-charge maternity care may in part cut across the social gradient, maternal unemployment remains an important public health issue in pregnancy even in the era of modern obstetric care. In summary, analysis of the observed data suggests that maternal unemployment is associated largely with social disadvantage, which results in increased risks when pregnant or in labor. The results clearly convey the impression that the principal reason for the association between a woman's unemployment and adverse pregnancy outcome is the presence of a series of correlated risk factors. However, correction for confounding factors did not entirely explain the association between unemployment and adverse pregnancy outcome. This was particularly the case for families with both parents unemployed but also when only the pregnant women was unemployed, and therefore effective measures should be considered.

## Competing interests

The author(s) declare that they have no competing interests.

## Authors' contributions

All authors (K.R., N.H., and S.H.) participated in designing the study, analyzing the results and writing the manuscript, K.R. coordinated the study. All authors read and approved the final manuscript.

## Pre-publication history

The pre-publication history for this paper can be accessed here:



## Figures and Tables

**Table 1:** Maternal Risk Factors in study groups I-III compared with the reference group

Risk factor	Reference (n = 18963) %	I Mother unemployed (n = 3388) %	P	II Father unemployed (n = 1551) %	P	III Both parents unemployed (n = 1037) %	P
Age < 18 years	0.4	1.3	0.001	1.81	0.001	2.51	0.001
Age > 35 years	12.9	7.9	0.001	8.90	0.001	5.79	0.001
Unmarried	27.4	43.1	0.001	45.0	0.001	58.0	0.001
Primiparity	40.3	41.7	0.12	45.8	0.001	49.8	0.001
Previous miscarriage	17.2	17.2	0.92	14.8	0.015	13.8	0.005
Prior termination	9.3	12.5	0.001	13.0	0.001	15.4	0.001
7 deliveries	0.5	0.06	0.001	0.4	0.59	0	0.01
IUD before pregnancy	9.02	7.29	0.001	6.25	0.001	6.56	0.006
Surgically scarred uterus	10.6	10.8	0.75	10.1	0.54	7.8	0.004
Second pregnancy in 12 months	6.9	7.5	0.24	8.6	0.014	8.3	0.089
Previous delivery > 6 y	9.7	9.1	0.30	8.1	0.040	8.8	0.35
Prior fetal demise	2.1	1.9	0.51	1.9	0.67	3.2	0.018
Low weight gain	6.8	9.1	0.001	7.6	0.34	9.0	0.02
Overweight	23.7	25.7	0.01	24.4	0.51	26.3	0.053
Smoking	4.2	9.5	0.001	10.4	0.001	17.9	0.001
Alcohol consumption	3.2	4.5	0.001	3.4	0.76	2.7	0.36
Chronical illness	5.7	6.2	0.26	5.5	0.83	6.3	0.42
Diabetes	2.0	2.8	0.003	2.2	0.58	2.9	0.044
Pregravid hypertension	1.9	2.2	0.24	2.0	0.69	2.1	0.54

IUD = intrauterine device

**Table 2:** Pregnancy and Delivery Characteristics in study groups I–III compared with the reference group

Characteristic	Reference (n = 18961) %	I Mother unemployed (n = 3388) %	P	II Father unemployed (n = 1551) %	P	III Both parents unemployed (n = 1037) %	P
Prolonged gravidity	4.9	5.3	0.38	4.1	0.12	5.1	0.79
Low hemoglobin	1.4	1.9	0.01	1.8	0.14	2.1	0.040
Meconium stained AF	10.5	11.4	0.11	10.4	0.99	11.1	0.51
Amnionitis	1.5	1.5	0.88	1.5	0.86	1.3	0.46
Pre-eclampsia	3.3	3.0	0.32	4.1	0.11	3.0	0.58
Inducted delivery	15.9	18.9	0.001	17.9	0.039	17.1	0.32

AF = amniotic fluid

**Table 3:** Pregnancy outcomes in study groups I–III compared with the reference group

Outcome	Group	%	Unadjusted OR	95% CI	Adjusted OR	95% CI
SGA	Reference	8.8				
	I Mother	10.8	1.26	1.11–1.41	1.26	1.12–1.42
	II Father	9.2	1.06	0.88–1.26	1.06	0.88–1.27
	III Both	14.0	1.69	1.41–2.03	1.43	1.18–1.73
						
Preterm delivery (< 37 weeks)	Reference	6.4				
	I Mother	6.2	0.98	0.84–1.14	0.97	0.83–1.13
	II Father	5.5	0.87	0.69–1.09	0.86	0.68–1.08
	III Both	6.8	1.08	0.84–1.39	0.99	0.77–1.29
						
Admission to neonatal unit	Reference	7.3				
	I Mother	7.5	1.03	0.89–1.18	1.03	0.90–1.19
	II Father	7.6	1.04	0.86–1.26	1.05	0.87–1.28
	III Both	7.6	1.04	0.82–1.32	1.0	0.78–1.27
						
Low Apgar score (< 7) 1 min	Reference	5.1				
	I Mother	4.9	0.97	0.82–1.15	0.95	0.80–1.12
	II Father	5.0	0.98	0.77–1.25	0.97	0.76–1.23
	III Both	4.6	0.91	0.68–1.23	0.79	0.58–1.08
						
Low Apgar score (<7) 5 min	Reference	1.9				
	I Mother	1.8	0.92	0.70–1.21	0.92	0.69–1.21
	II Father	2.0	1.04	0.72–1.50	1.02	0.70–1.48
	III Both	1.5	0.75	0.44–1.25	0.68	0.40–1.16
						
Fetal venous pH < 7.15 at birth	Reference	1.0				
	I Mother	1.2	1.20	0.86–1.68	1.20	0.86–1.69
	II Father	1.2	1.12	0.69–1.83	1.12	0.69–1.82
	III Both	1.5	1.41	0.83–2.38	1.03	0.68–1.54
						
Abnormal FHR during delivery	Reference	15.7				
	I Mother	14.0	0.87	0.79–0.97	0.86	0.77–0.96
	II Father	15.2	0.97	0.84–1.12	0.95	0.82–1.10
	III Both	16.5	1.06	0.90–1.26	0.93	0.79–1.12
						
Fetal death	Reference	0.4				
	I Mother	0.5	1.29	0.76–2.18	1.30	0.76–2.20
	II Father	0.4	0.99	0.43–2.28	1.04	0.45–2.39
	III Both	0.4	0.99	0.36–2.71	1.0	0.36–2.79
						
Neonatal death	Reference	0.2		0.40–1.98		
	I Mother	0.2	0.89	0.13–2.29	0.80	0.40–1.96
	II Father	0.1	0.56	0.60–4.64	0.56	0.13–2.30
	III Both	0.4	1.66		1.65	0.58–4.68

*OR adjusted for age, parity, smoking, alcohol consumption, infertility, abortions, previous fetal deaths and miscarriages, time since previous pregnancy and maternal illness

**Table 4:** Unadjusted Odds Ratios of pregnancy outcome in unemployed women compared to families where both parents are unemployed

Outcome	I Mother unemployed (%)	III Both parents unemployed (%)	Unadjusted OR (95% CI)
SGA	10.8	14.0	1.35 (1.10–1.65)
Preterm delivery	6.2	6.8	1.10 (0.83–1.46)
Admission to neonatal unit	7.5	7.6	1.01 (0.78–1.32)
Low Apgar score (< 7) 1 min	4.9	4.6	0.94 (0.68–1.31)
Low Apgar score (<7) 5 min	1.8	1.5	0.81 (0.46–1.44)
Fetal venous pH < 7.15 at birth	1.2	1.5	1.17 (0.65–2.12)
Abnormal FHR during delivery	14.0	16.5	1.22 (1.01–1.47)
Fetal death	0.5	0.4	0.77 (0.26–2.29)
Neonatal death	0.2	0.4	1.87 (0.55–6.40)

FHR = fetal heart rate
